# Evaluation of *p*-cresol degradation with polyphenol oxidase (PPO) immobilized in various matrices

**DOI:** 10.1007/s13205-016-0547-y

**Published:** 2016-10-26

**Authors:** Vijayalakshmi A. Edalli, Sikandar I. Mulla, Syed Ali Musstjab Akber Shah Eqani, Gurumurthy D. Mahadevan, Rohit Sharma, Yogesh Shouche, Chandrappa M. Kamanavalli

**Affiliations:** 1 Department of Biochemistry, Karnatak University, Dharwad, Karnataka 580 003 India; 2 Key Laboratory of Urban Environment and Health, Institute of Urban Environment, Chinese Academy of Sciences, Xiamen, 361021 China; 3 Key Laboratory of Urban Pollutant Conversion, Institute of Urban Environment, Chinese Academy of Sciences, Xiamen, 361021 China; 4 National Center for Cell Science, University of Pune, Ganeshkhind, Pune, 411007 India

**Keywords:** Silver nanoparticles (AgNPs), Polyphenol oxidase (PPO), *p*-Cresol, Biodegradation, *Pleurotus* sp. isolate VLECK02

## Abstract

*p*-Cresol is an environmental pollutant due to its vast use, toxicity and persistence, nevertheless, its degradation in an enzyme is unclear. In this study, we used *Pleurotus* sp. isolate VLECK02 polyphenol oxidase (PPO) for the determination of *p*-cresol degradation. On the basis of UV, FT-IR and chromatographic (HPLC and GC–MS) analysis, 4-methylcatechol was identified as the main metabolite of *p*-cresol catabolism. In addition, batch and semi-continuous degradation of *p*-cresol (10 and 20 mM) were studied and compared by free and immobilized PPO in different matrices like sodium alginate (SA), sodium alginate–polyvinyl alcohol (SA–PVA) and sodium alginate–polyvinyl alcohol–silver nanoparticles (SA–PVA–AgNPs). The experimental data showed that an enzyme (PPO) immobilized in SA–PVA–AgNPs was completely degraded *p*-cresol at initial concentrations of 10 and 20 mM within 30 h. These results suggest that the enzyme immobilized in SA–PVA–AgNPs has achieved higher degradation rates at a given time than free PPO and PPO immobilized in SA–PVA and SA. The SA–PVA–AgNPs and SA–PVA immobilized enzyme could be reused for more than 12 and 8 cycles, respectively, without losing any degradation capacity. Moreover, the immobilized PPO showed higher tolerance to various temperatures and pH than free PPO. Hence, immobilized PPO could be useful for the bioremediation of environment contaminated with phenolic compounds like *p*-cresol.

**Electronic supplementary material:**

The online version of this article (doi:10.1007/s13205-016-0547-y) contains supplementary material, which is available to authorized users.

## Introduction

Due to the higher water solubility, certain organic compounds like cresol or oil contained phenol mélanges and some mono-aromatic hydrocarbons were considered as principle contaminants in groundwater (Flyvbjerg et al. 1993; Rosenfeld and Plumb 1991). *p*-Cresol or 4-methylphenol [CH_3_C_6_H_4_(OH)] is an aromatic compound of phenol derivatives and is generally extracted from coal–tar in coal gasification plants by fractionation and various other synthetic processes (Muller et al. 2001). *p*-Cresol is a toxic chemical, corrosive in nature, causes nervous system depression and is also a common by-product produced from tyrosine by several anaerobic organisms (Tallur et al. 2006). Hence, it is listed as a significant contaminant by the US Environmental Protection Agency (Tallur et al. 2006).

There are reports on removal/degradation of *p*-cresol by enzymes, microorganisms and immobilized cells of organism(s) (Gunther et al. 1995; Kolomytseva et al. 2007; Muller et al. 2001; Singh et al. 2008; Tallur et al. 2006, 2009; Vermette 2000; Yamada et al. 2007). However, several disadvantages in the direct microbial detoxification of pollutants were determined. The influence of physicochemical and poor bioavailability of contaminants, the presence of other toxic compounds on microbial growth will directly affect detoxification (Karigar and Rao 2011). Hence, the enzymatic process has an advantage than microorganisms by overcoming these problems for the detoxification of toxic pollutants and is useful for industrial applications. There are reports on copper-containing enzymes like polyphenol oxidases (PPO) which catalyze the oxidation of phenol derivatives in the presence of O_2_ (Burton 1994; Rapeanu et al. 2006) and also used for the detoxification of other organic contaminants (Bollag et al. 2003; Hou et al. 2011; Rodriguez Couto and Toca Herrera 2006). However, PPOs from different sources might differ in substrate specificity and catalytic competency (Durán et al. 2002). Application of immobilization technology will help in improving the stability of enzyme for the enhanced detoxification of toxic pollutants and fermentation process. Various matrices are used in enzyme immobilization such as chitosan microspheres, polymeric carrier, polyacrylonitrile beads, magnetic chitosan nanoparticles, nano-porous silica beads and alginate-SiO_2_ hybrid gel (Dehghanifard et al. 2013; Jiang et al. 2005; Kalkan et al. 2012; Nicolucci et al. 2011; Shao et al. 2009; Stanescu et al. 2012). Although, many previous studies used immobilized PPOs for the removal of textile dyes, non-textile dyes, aqueous phenol and phenol derivatives (Arabaci and Usluoglu 2014; Khan and Husain 2007; Lončar et al. 2011; Shao et al. 2009), yet, there is not much information available on oxidation of *p*-cresol by immobilized PPO. With this background, the aim of this study is to explore the possibility of *p*-cresol degradation by *Pleurotus* sp. isolate VLECK02 PPO immobilized on various matrices like sodium alginate (SA), sodium alginate–polyvinyl alcohol (SA–PVA) and SA–PVA–silver nanoparticles (AgNPs).

## Materials and methods

### Materials

The mushroom was obtained from University of Agricultural Sciences (UAS), Dharwad, Karnataka State, India. Aniline, 2-chloroaniline, 3-chloroaniline, 4-chloroaniline, *p*-cresol, catechol and 4-methylcatechol with highest purity were procured from Merck and Sigma-Aldrich. All other chemicals used in this study were of analytical grade.

### Maintenance and cultivation of mushroom culture

Mushroom mycelium was grown on standard YpSs agar plate at 45 °C until sporulation and stored in an incubator at 20 °C. Stock culture was sub-cultured for every 3 weeks and was used for the inoculation of pre-cultures.

### Identification of fungus (mushroom culture) by 18S rRNA gene sequence analysis

Isolation of DNA and 18S rRNA gene sequence analysis was carried out according to the method described in Singh et al. (2010). PCR amplification of 18S and ITS regions of rDNA partial region of SSU rDNA was amplified by PCR using universal fungal primers, NS1 (5′-GTAGTCATATGCTTGTCTC-3′) and NS4 (5′-CTTCCGTCAATTCCTTTAAG-3′) of ∼1100 bp. Full length of ITS region of ∼600 bp was amplified using the primers ITS1 (5′-TCCGTAGGTGAACCTGCGG-3′) and ITS4 (5′-TCCTCCGCTTATTGATATGC-3′) (Bagewadi et al. 2016). The conditions for PCR included an initial hot start incubation (5 min at 94 °C) followed by 34 cycles (denaturation at 94 °C for 30 s, annealing at 55 °C for 30 s and extension at 72 °C for 1 min) and a final extension at 72 °C for 5 min. Fresh PCR products were purified by using gel extraction kit (Sigma, Genosys, USA) and sequenced using the Big Dye Terminator cycle sequencing kit (V3.1, Applied Biosystems, USA) according to the manufacturer’s protocol and analyzed in a DNA Analyzer (3730 DNA Analyzer, Applied Biosystems, USA). Sequence data were edited using Chromas Pro version 1.34. Fungal rDNA −18S and ITS sequence in this study and the matched sequences from Ez-Taxon and GenBank data libraries were analyzed using BLAST-N program and aligned by Clustal-W. The phylogenetic analysis with 1000 bootstrap replicates was conducted by MEGA 6 software using neighbor-joining method. The nucleotide sequence was identified as *Pleurotus* sp. isolate VLECK02 and deposited in National Centre for Biotechnology Information (NCBI) GenBank with Accession Number KU752353.

### Extraction, purification and quantification of an enzyme, polyphenol oxidase (PPO)

Mushroom polyphenol oxidase (PPO) was extracted according to the method described previously (Bevilaqua et al. 2002; Edalli and Kamanavalli 2010). The collected supernatant was precipitated with ammonium sulfate at 35–80% saturation for 1 h with gentle stirring. The precipitate was collected by centrifugation at 15,000×*g* for 20 min. The precipitate was redissolved in 50 mM phosphate buffer (pH 7.0) and dialyzed at 4 °C against 10 mM phosphate buffer (pH 7.0). Approximately, 10 mL of clear extract was applied to Sephadex G-100 column (1.2 × 70 cm, 0.5 mL/min). Fractions (2 mL) were collected and tested for PPO activity and protein. Further purification was carried out with DEAE-cellulose anion exchange column (1.2 × 70 cm, 1 mL/min). Fractions containing PPO activity were pooled, dialyzed and concentrated (Flurkey et al. 2008; Hamed et al. 2008).

PPO activity was determined using catechol as a substrate. The total reaction mixture volume (2 mL) containing 1 mL of 0.2 M phosphate buffer (pH 7.0), 0.5 mL of culture supernatant and 0.5 mL of 10 mM catechol in 0.2 M phosphate buffer at pH 7.0 was incubated at 50 °C for 3 min and the change in absorbance at 420 nm was measured spectrophotometrically. One unit of enzyme (PPO) activity was defined as the amount of enzyme that increased absorbance of 0.001 per minute (Bevilaqua et al. 2002; Hou et al. 2011).

### Degradation of *p*-cresol by PPO and identification of metabolites

The effects of initial concentration of *p*-cresol (10–20 mM) on PPO were examined at the optimum pH. For *p*-cresol degradation, mushroom PPO (2 mL, 1220 units) was added to 50 mL *p*-cresol solution (10 mM in 50 mM phosphate buffer, pH 7.0) and stirred, the reaction mixture gradually turned to brown due to degradation of *p*-cresol. After 6 h, the brown precipitate formed was separated by centrifugation (10,000×*g* for 15 min) and the supernatant was acidified to pH 2.0 with 1 N HCl and extracted twice with diethyl ether, dehydrated (passed through anhydrous sodium sulfate), dried and dissolved in methanol (Tallur et al. 2006). After filtration with 0.45 µm membrane, the samples were analyzed by UV, FT-IR, HPLC and GC–MS.

### Immobilization of PPO in different matrices

#### Sodium alginate (SA) entrapment

Mushroom PPO was immobilized on SA entrapment according to the method described previously (Tallur et al. 2015). To 20 mL enzyme preparation, 400 mg of SA was added so as to obtain a 2% solution of SA. This solution was then extruded dropwise into sterile, cold 0.2 M CaCl_2_ solution through burette connected to a tapered pipette tip. Gel beads of approximately 2 mm diameter were obtained. The gel beads were hardened by re-suspending into a fresh calcium chloride for 2 h with gentle agitation. Finally, these beads were washed with sterilized distilled water for several times and stored in 0.1 M phosphate buffer (pH 7.0) for further use.

#### Sodium alginate–polyvinyl alcohol (SA–PVA) gel entrapment

The SA–PVA entrapment of mushroom PPO was performed according to the method described in Mulla et al. (2012). Aqueous solutions of 5% (w/v) of polyvinyl alcohol and 2% (w/v) of sodium alginate were mixed at the ratio of 60:40. In this mixture, 20 mL of enzyme preparation was dispersed. Glutaraldehyde was used as a crosslinker. The hydrogel spheres were prepared by dispensing the mixture dropwise manner with 10 mL pipette into 0.2 M CaCl_2_ solution. The beads were washed with water and stored in 0.1 M phosphate buffer (pH 7.0).

#### Entrapped in sodium alginate–polyvinyl alcohol–silver nanoparticles (SA–PVA–AgNPs) gel

An aqueous solution of SA–PVA was prepared as described above. To this, AgNO_3_ (2 × 10^−4^ M) was added gently, stirred in an ice-water bath until the solution becomes colorless and then 10 mL of NaBH_4_ (4 × 10^−4^ M) aqueous solution was added dropwise under vigorous stirring at the same temperature for 2 h. The solution became light yellow in color (Eqs. 1 and 2). To this, 20 mL of the enzyme was added, and hydrogels were prepared by dispensing the mixture dropwise manner with 10 mL pipette into 0.2 M CaCl_2_ solution. The beads were washed with water and stored in 0.1 M phosphate buffer (pH 7.0) (Mbhele et al. 2003; Shameli et al. 2010).

1 $$ \left( {n/8} \right){\text{BH}}_{4}^{ - } + \, n{\text{Ag}}^{ + } + \, \left( {n/2} \right){\text{ H}}_{2} {\text{O }} \to {\text{ Ag}}_{n} + \, \left( {n/8} \right) \, {\text{B}} \, \left( {\text{OH}} \right)_{4}^{ - } + n{\text{H}}^{ + } $$

2 $$ {\text{BH}}_{4}^{ - } + \, 4{\text{ H}}_{2} {\text{O }} \to {\text{ H}}_{3} {\text{BO}}_{4} + {\text{ OH}}^{ - } + \, 4{\text{H}}_{2} $$

### Batch degradation and stability experiments

The batch degradation experiments were performed to evaluate the degradation of *p*-cresol by free and immobilized enzyme in different matrices. To 100 mL of *p*-cresol solution (10 and 20 mM in 50 mM phosphate buffer, pH 7.0), 2 mL of enzyme preparation was added along with control. In separate flasks, 10 g wet beads (approximately 500 beads) of various matrices, were added to 100 mL of *p*-cresol solution. Biodegradation experiment was carried out on a rotary shaker (150 rpm) to allow continuous oxygenation. The samples were taken out at regular intervals for the analysis of *p*-cresol by high-performance liquid chromatography (HPLC). The degradation of *p*-cresol by immobilized and free enzyme (PPO) at different temperature (10–50 °C) and pH (6.0–10) were also studied.

To determine the stability of immobilized enzyme on *p*-cresol degradation, repeated batch degradation experiments were conducted. After each cycle of incubation (30 h/cycle), the spent solution was decanted and the beads were washed with sterile water and a fresh solution of *p*-cresol was added. The process was carried out under identical conditions and spent solution was analyzed for the residual of *p*-cresol by HPLC.

### Analytical methods

The *p*-cresol concentration in the spent solution was determined by HPLC (Tallur et al. 2006). At regular intervals, the spent solution was collected and centrifuged at 10,000×*g*. The supernatants were then analyzed by reversed phase HPLC with 5-µ sperisorb-ODS (C_18_ column). Acetonitrile and potassium phosphate buffer (50 mM, pH 7) solvent system at a ratio of 60:40 (v/v) were used as the mobile phase. The flow rate was kept at 1 mL/min. The residual (*p*-cresol) and by-product peaks were determined at 278 and 272 nm, respectively. The *p*-cresol metabolites were analyzed by GC–MS Shimadzu QP2010 Plus (Mulla et al. 2011). The temperature program was held at 50 °C for 1 min with 15 °C increase/min to a final temperature 280 °C for 15 min and the injector temperature was kept at 250 °C. 1 µL volume was used for the injection and helium was used as a carrier gas. Electron ionization energy of 70 eV was used to operate the mass spectrometer. Further, the metabolites were also confirmed by UV–visible spectrophotometer and Nicolet Impact 410 FT-IR (Mulla et al. 2011).

## Results

### Identification of fungal (mushroom) species by 18S rDNA gene sequence analysis

**Fig. 1:**
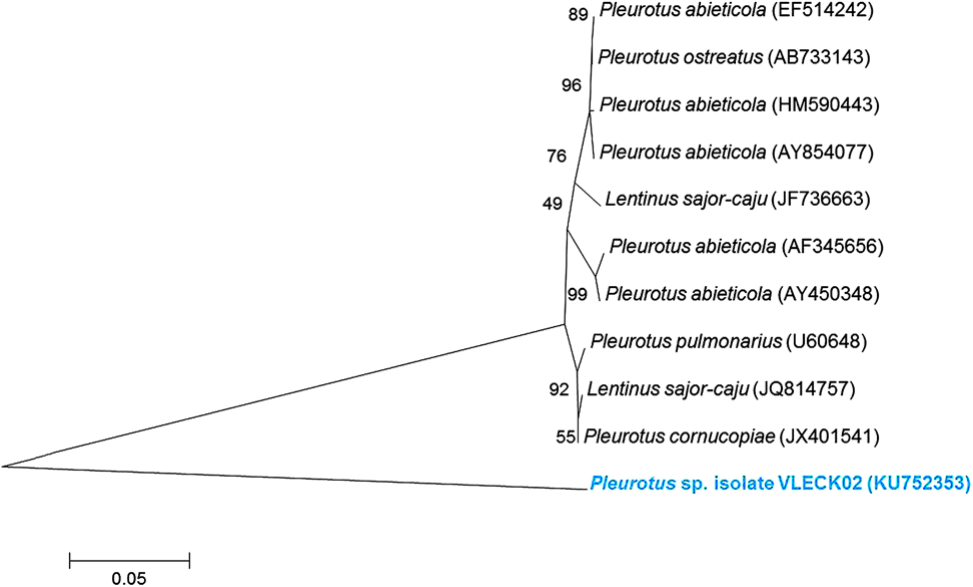
Phylogenetic relationships established based on 18S rRNA gene sequences of fungal strain (*Pleurotus* sp. isolate VLECK02, KU752353). The organism sequence was used for BLAST analysis in ez-Taxon and the nearest neighbor sequences of other fungal cultures were chosen for phylogenetic tree construction using MEGA 6 software with neighbor-joining method. *Numbers at branches* are bootstrap values of 1000 replications

18S rDNA gene sequence analysis was done on the basis of the sequence difference present in the ITS region. The sequence data obtained were then aligned to identify the closest homologs using Ez-Taxon database. The phylogenetic tree (Fig. 1) was constructed by the neighboring-joining (NJ) method with other related *Pleurotus* strains from the Ez-Taxon. Further, the organism was designated as *Pleurotus* sp. isolate VLECK02. The ITS sequence has been deposited in NCBI GenBank under the accession number KU752353.

### Enzyme extraction and quantification

During the enzyme extraction and purification, several precipitations with solid ammonium sulfate between 35 and 80% were tested to know the proper saturation point. As a result, the PPO activity of the precipitate of 65% (NH_4_)_2_SO_4_ saturation was found to be highest and this saturation point was used in all the extraction processes. The results of specific activities and purification degrees of PPO of mushroom *Pleurotus* sp. isolate VLECK02 was described in Table 1.

**Table 1:** Purification of PPO extracted from *Pleurotus* sp. isolate VLECK02

Steps	Activity (units/mL/min)	Protein (mg/mL)	Specific activity (units/mL/mg of protein)	Yield (%)	Fold purification
Crude extract	1220	3.52	346.59	100	1.0
30% NH_4_ (SO_4_)	810	2.08	389.423	59.09	1.12
65% NH_4_ (SO_4_)	2152	1.52	1415.789	43.18	4.08
Gel filtration	2805	0.64	4382.81	18.18	12.65
Ion exchange	3540	0.395	8962.02	11.22	25.86

### Effect of temperature and pH on free and immobilized PPO

**Fig. 2:**
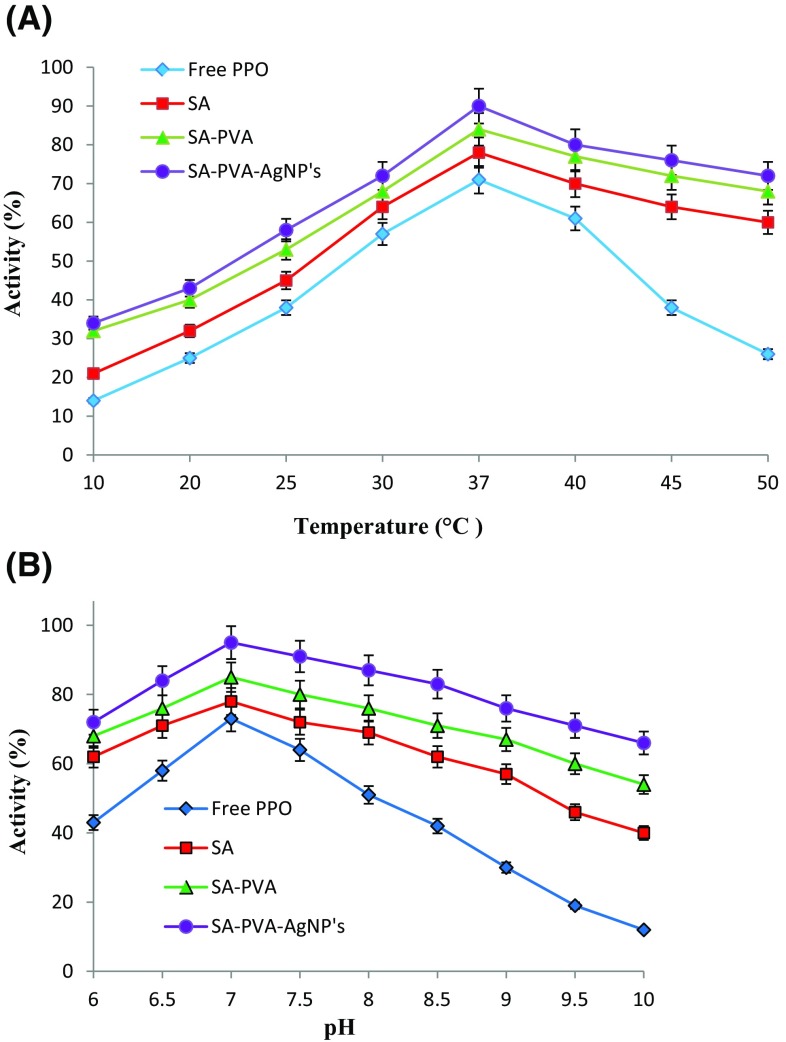
Effect of temperature (**a**) and pH (**b**) on activity of free and immobilized PPO. The optimum temperature and pH on activity of free and immobilized PPO was considered as 100%. Experiments were performed in triplicate and *error bars* represent standard deviation

Activity dependence of free and immobilized PPO on temperature and pH were studied. When compared to free PPO, the SA–PVA and SA–PVA–AgNPs immobilized PPO has shown enhanced activity at higher temperatures between 30 and 50 °C. In brief, the immobilized PPO in SA–PVA and SA–PVA–AgNPs at 50 °C retains about 72 and 68% residual activity, respectively. On the other hand, free and in SA immobilized PPO, the activity was lowered to 26 and 60%, respectively (Fig. 2a). The optimum activity for free and immobilized PPO was determined using 50 mM phosphate buffer (pH 6.0–10). The optimum activity for free and immobilized PPO was determined at pH 7.0, whereas the variation of initial pH from 6 to 9 had no effect on the oxidation of *p*-cresol by the immobilized enzyme. On the other hand, free PPO showed higher oxidation rate at pH 7.0 (Fig. 2b).

### Characterization of surface morphologies of polymer beads

Figure S1 (Supplementary Information, SI) illustrate the scanning electron micrograph of PPO immobilized SA–PVA and SA–PVA–AgNPs polymer beads. The PPO immobilized SA–PVA–AgNPs beads were show more compact structure and significantly different surface morphology when compared with SA–PVA. The micrographs confirm that by the addition of AgNPs, there is a uniform distribution of particles throughout the membrane matrix with no apparent clustering. This ensures that the AgNP-incorporated membranes obtained here were free from possible defects and which increases the membrane stability.

### Degradation of *p*-cresol by free and immobilized enzyme in batch experiments

**Fig. 3:**
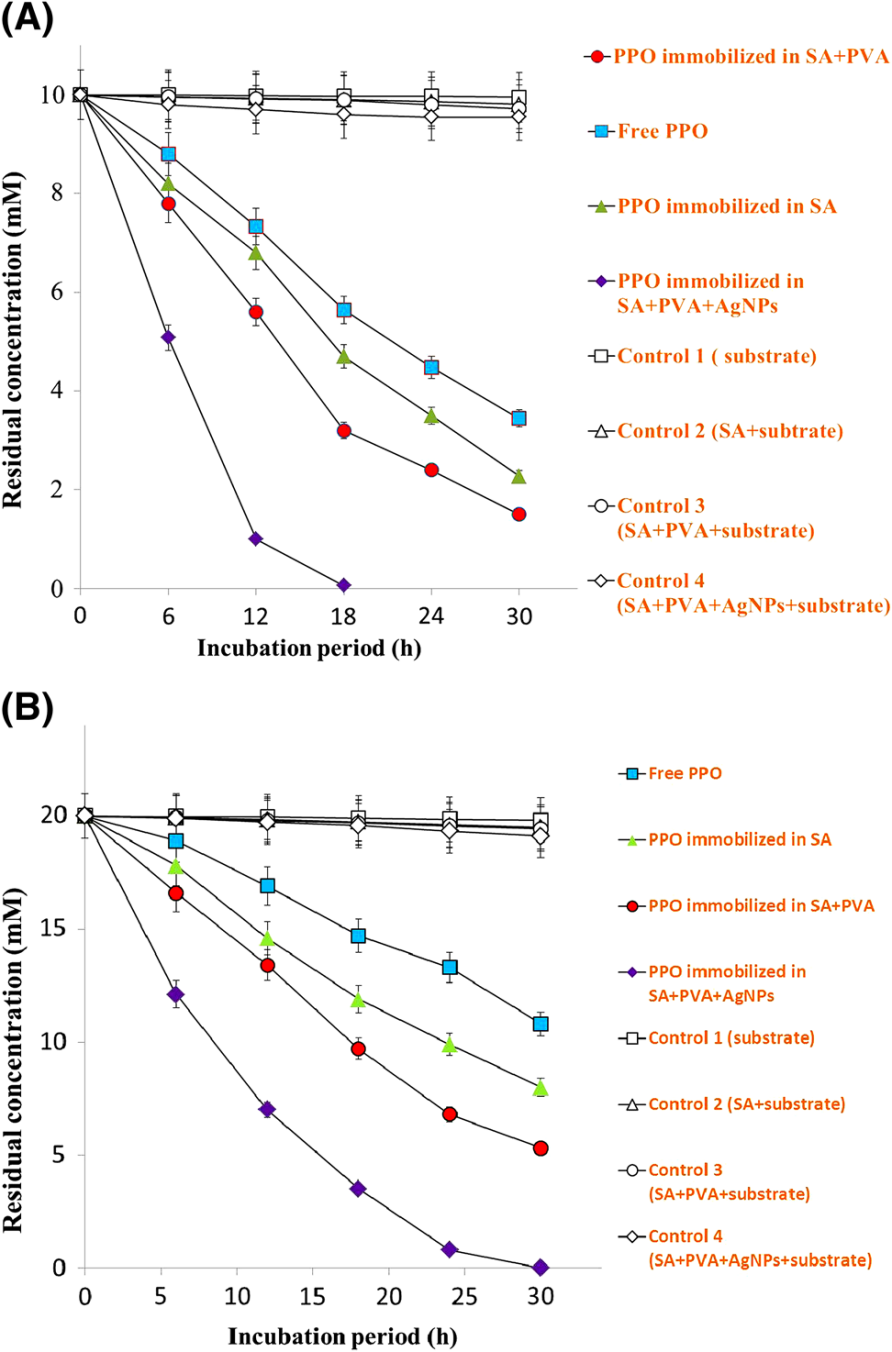
Oxidation of *p*-cresol at 10 mM (**a**) and 20 mM (**b**) concentration in batch process by PPO immobilized in SA–PVA–AgNPs, SA–PVA, SA, free enzyme and controls (*1*–*4*). Experiments were performed in triplicate and *error bars* represent standard deviation

The degradation of 10 and 20 mM of *p*-cresol was carried out in batch experiments by free PPO [2 mL (1220 units/mL) enzyme preparation in 100 mL *p*-cresol solution], and PPO immobilized in SA, SA–PVA and SA–PVA–AgNPs (10 g wet beads, the enzyme concentration was in the range of 220, 208 and 198 units/2 g beads for SA–PVA–AgNPs, SA–PVA and SA, respectively) and their results are given in Fig. 3a, b. Free enzyme degraded 85% of *p*-cresol after 30 h of incubation from an initial concentration of 10 mM, whereas only 49% was removed when the initial concentration was increased to 20 mM. The enzyme immobilized in SA–PVA–AgNPs completely degrades 10 and 20 mM of *p*-cresol within 30 h. The enzyme immobilized in PVA–SA and SA degraded 77 and 65%, respectively. However, in controls (1–4), *p*-cresol was adsorbed/degraded at minute level (Fig. 3a, b).

### Stability of immobilized enzyme for degradation of *p*-cresol

**Fig. 4:**
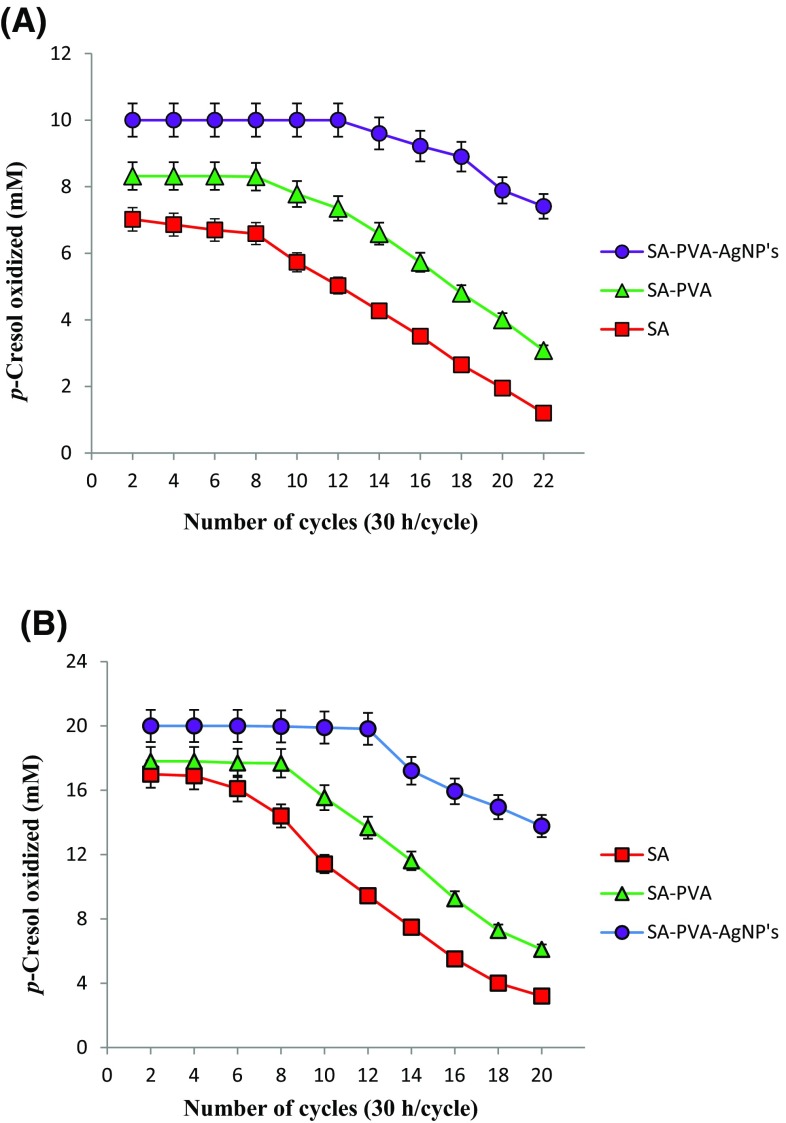
Oxidation of *p*-cresol at 10 mM (**a**) and 20 mM (**b**) concentration in repeated batch process by PPO immobilized in SA–PVA–AgNPs, SA–PVA, SA. Experiments were performed in triplicate and *error bars* represent standard deviation

The repeated batch degradation of *p*-cresol by enzyme immobilized in SA, SA–PVA and SA–PVA–AgNPs (10 g wet beads) was carried out at two different concentration (10 and 20 mM) for 30 h and their results are shown in the Fig. 4a, b. The enzyme immobilized in SA–PVA–AgNPs and SA–PVA could be reused for 12 and 8 cycles, respectively, without losing the degradation capacity with both the concentrations of *p*-cresol (Fig. 4a, b). However, the enzyme immobilized in SA could be reused for eight cycles and after that beads were slowly degraded in the solution with 10 mM (Fig. 4a). When the initial concentration was increased to 20 mM, SA immobilized beads were reused with decreased rate of oxidation of *p*-cresol (Fig. 4b).

### Identification of metabolites during degradation of *p*-cresol by PPO

**Fig. 5:**
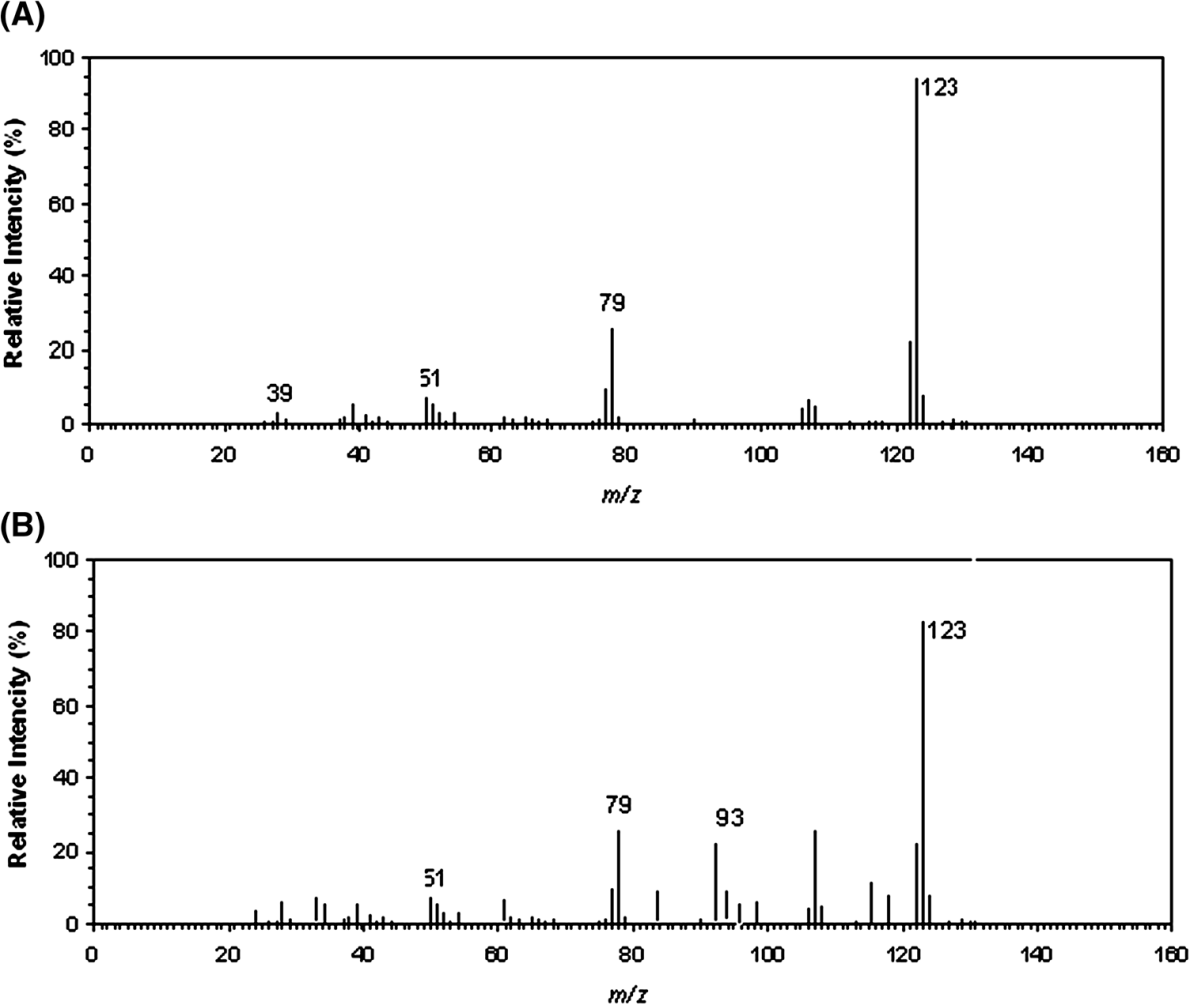
Mass spectrum of isolated metabolite of *p*-cresol oxidation by PPO (*Pleurotus* sp. isolate VLECK02) (**a**) and the authentic compound 4-methylcatechol (**b**)

For the identification of metabolites during degradation of *p*-cresol (10 mM) by PPO, the samples were collected at different time and the intermediates from reaction mixture were analyzed by UV, HPLC, FT-IR, and GC–MS. The UV spectrum of metabolite showed absorption maximum at 272 nm identical that of authentic 4-methylcatechol (Fig. S2, SI). Figure S3 (SI) illustrate the presence of metabolite (peak) with the retention time of 2.88 min, while for 4-methylcatechol standard solution, a peak was detected at a similar interval. The IR spectrum showed characteristic absorption bands of –OH stretching at 3348.5 cm^−1^, C–O stretching at 1281 cm^−1^, C=O stretching at 1674 cm^−1^, aromatic CH stretching at 2924.7 cm^−1^ and >C=C< stretching at 1658.8 cm^−1^ (Fig. S4, SI). The mass spectrum of isolated metabolite (Fig. 5a) was in good agreement with that of authentic 4-methylcatechol (Fig. 5b).

## Discussion

From the above results, it is confirmed that 4-methylcatechol was accumulated during the oxidation of *p*-cresol by PPO (*Pleurotus* sp. isolate VLECK02). Microbes play an essential role in detoxification of *p*-cresol which proceeds through two different routes. In some organisms, the aromatic ring of *p*-cresol was hydroxylated to form 4-methylcatechol whereas in some other cases the *p*-cresol containing methyl group was oxidized into 4-hydroxybenzoic acid which further transformed into protocatechuate (Kolomytseva et al. 2007; Tallur et al. 2006).

Additionally, in batch degradation, the data obtained from immobilized PPO in SA–PVA–AgNPs revealed that the rate of degradation of *p*-cresol even at higher concentration (20 mM) was much higher than that of free enzyme. The increased degradation by immobilized PPO was due to the accelerated activity of PPO by AgNPs. Therefore, the addition of AgNPs to SA–PVA matrix significantly enhanced the degradation of *p*-cresol and complete degradation was achieved within 18 h. Immobilization provides a kind of membrane protection, which might be responsible for the stabilization of enzyme activity and better degradation rates in the immobilized enzyme (Mulla et al. 2012; Hoskeri et al. 2014; Tallur et al. 2015). The surface morphology of PPO immobilized in SA–PVA–AgNPs bead shows more compact structure when compared with the surface of SA–PVA immobilized beads. Due to the compact surface structure enzyme released slowly and stability of enzyme also increases. The study of the effect of different metal ions on the activity of PPO showed that Ag ions and AgNPs enhance the PPO activity approximately 2–3 times. Therefore, we used AgNPs for PPO immobilization, which significantly enhanced the degradation of *p*-cresol.

The results of repeated batch degradation revealed that SA–PVA–AgNPs and SA–PVA immobilized PPO retained their *p*-cresol degrading ability for longer periods and could be reused for 12 and 8 cycles, respectively. The storage stability and activity of the enzyme in SA–PVA–AgNPs was better than that of encapsulated in other matrices. The SA entrapped PPO showed that with increased number of cycles, the rate of degradation decreased, due to gradual leakage of PPO from these beads (Tallur et al. 2015). The thermostability of immobilized PPO enzyme was apparently superior to that of free enzyme. Furthermore, both immobilized and free PPO gave similar pH values. However, the immobilized PPO offered much wider pH stability than free PPO. These results suggests that the enzyme (PPO) immobilized in various matrices were more resistant to different pH than free enzyme. The higher tolerance of temperature and pH of enzymes arising from immobilization would be a greater benefit to its industrial application due to the wide range of temperature and pH used in the industrial processes. Therefore, PPO can be used for the detoxification of cresol contaminated sites.

## Electronic supplementary material

Below is the link to the electronic supplementary material.

Supplementary material 1 (DOCX 1198 kb)
