# Supplementary material for: Evaluation of p-cresol degradation with polyphenol oxidase (PPO) immobilized in various matrices
Source: 3 Biotech. 2016 Oct 26;6(2):229. doi: 10.1007/s13205-016-0547-y (PMC5082039; doi:10.1007/s13205-016-0547-y)
Supplement: Supplementary file 1 — Supplementary material 1 (DOCX 1198 kb) [file 13205_2016_547_MOESM1_ESM.docx]

**Supplementary Information**

**Evaluation of *p*-cresol degradation with polyphenoloxidase (PPO) immobilized in various matrices**

Vijayalakshmi A. Edalli^a,^*, Sikandar I. Mulla^a^, Syed Ali Musstjab Akber Shah Eqani^b^, Gurumurthy D. Mahadevan^c^, Rohit Sharma^d^, Yogesh Shouche^d^ and Chandrappa M. Kamanavalli^a,^**

^a^Department of Biochemistry, Karnatak University, Dharwad-580 003, Karnataka, India.

^b^Key Laboratory of Urban Environment and Health, Institute of Urban Environment, Chinese Academy of Sciences, Xiamen, 361021, China.

^c^Key Laboratory of Urban Pollutant Conversion, Institute of Urban Environment, Chinese Academy of Sciences, Xiamen, 361021, China.

^d^National Center for Cell Science, University of Pune, Ganeshkhind, Pune 411007, India.

**Running title Degradation of *p*-cresol by immobilized PPO**

***Correspondence to:** Mrs. Vijaylaxmi A. Edalli (Ph.D.,)

(Responsible for manuscript submission),

Department of Biochemistry,

Karnatak University,

Dharwad – 580 003, India

Email: vijuedalli@gmail.com

Tel. Phone: +91-836-2215243; Fax No.: +91836-2747884

****Correspondence to:** Dr. C M. Kamanavalli,

Department of Biochemistry,

Karnatak University,

Dharwad – 580 003, India

Email: cmkamanavalli@gmail.com

Tel. Phone: +91-836-2215243; Fax No.: +91836-2747884

**Supporting Information: 5 Pages, 4 Figures**


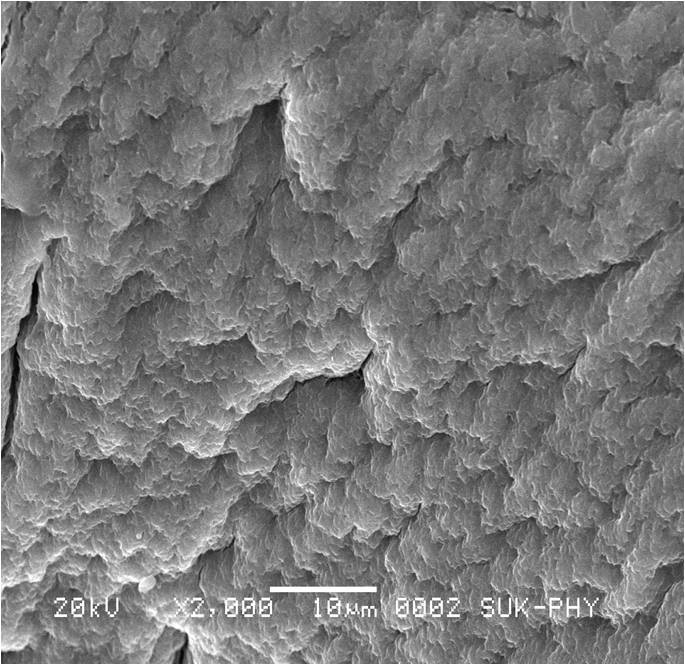


**A**


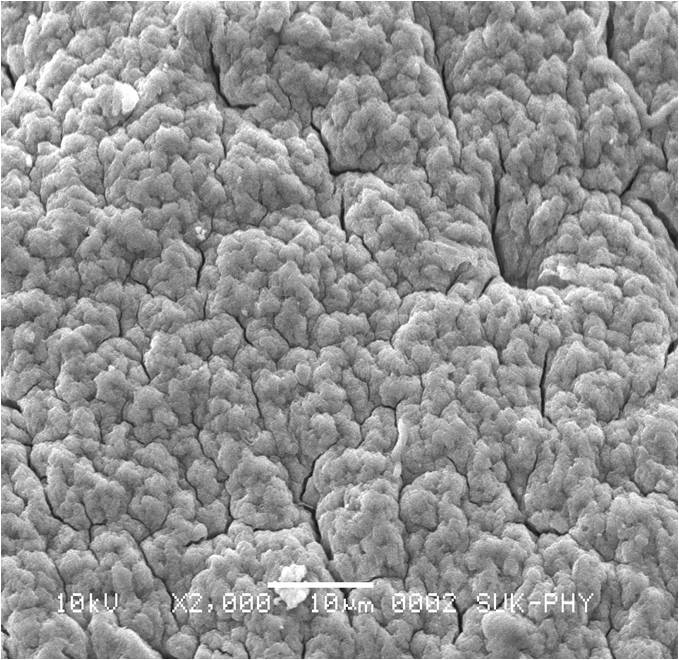


**B**

**Fig. S1** SEM micrographs of PPO immobilized polymer beads of SA-PVA (A) and SA-PVA-AgNPs (B).

**Fig. S2** UV spectrum of isolated metabolite (A) and the authentic compound 4-methylcatechol (B).


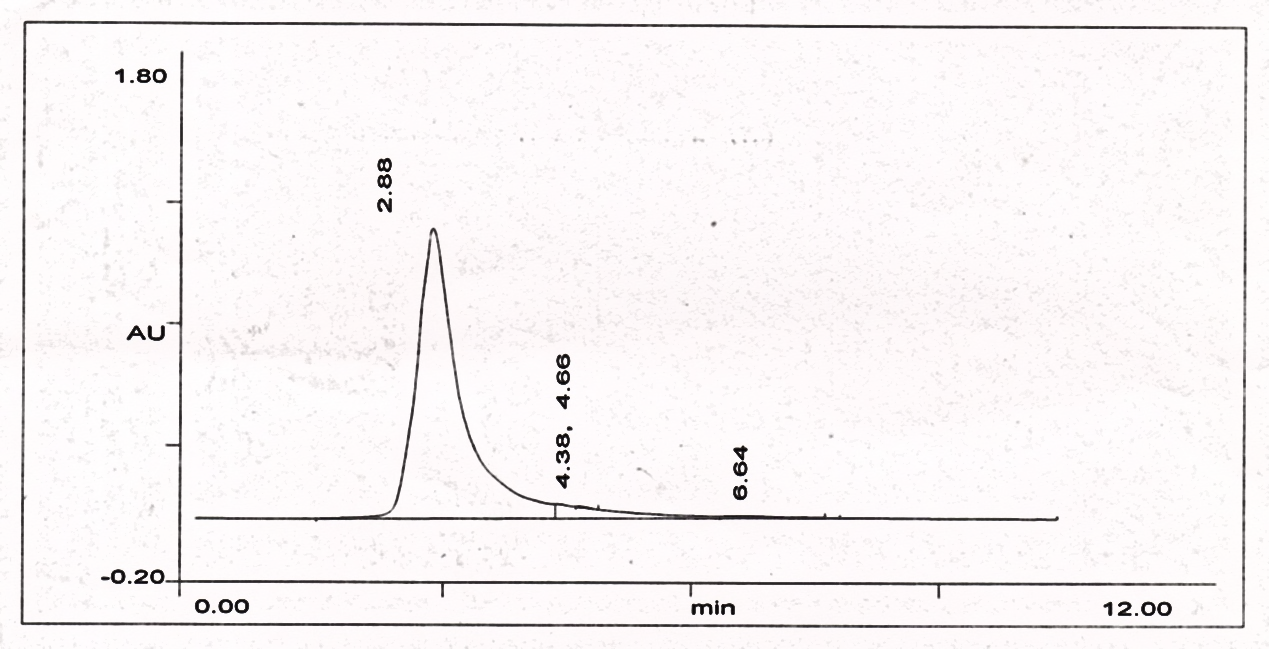


**(A)**


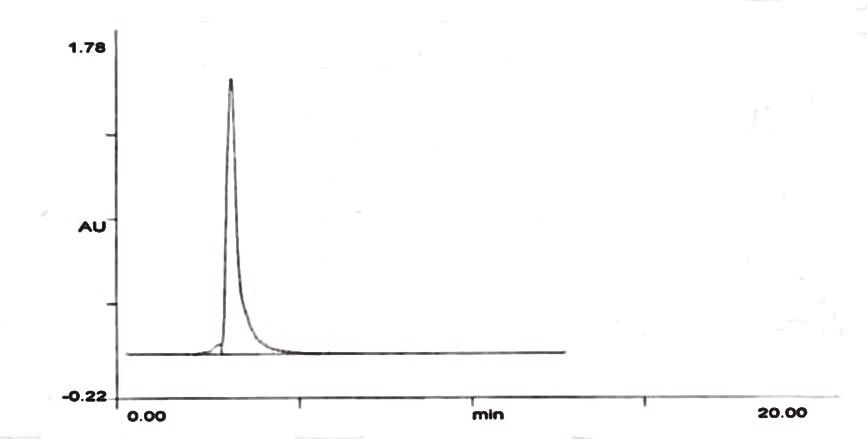


**(B)**

**Fig. S3** HPLC chromatogram of isolated metabolite (A) and the authentic compound 4-methylcatechol (B).

**
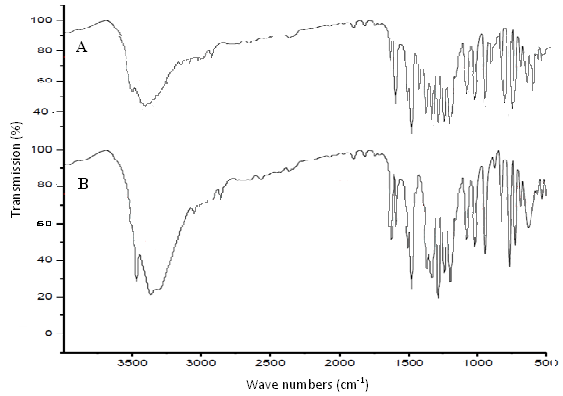
**

**Fig. S4** IR spectrum of isolated metabolite (A) and the authentic compound 4-methylcatechol (B).
